# Distinguishing the effects of systemic CSF1R inhibition by PLX3397 on microglia and peripheral immune cells

**DOI:** 10.1186/s12974-023-02924-5

**Published:** 2023-10-21

**Authors:** Akhabue K. Okojie, Joseph O. Uweru, Morgan A. Coburn, Sihan Li, Vivian D. Cao-Dao, Ukpong B. Eyo

**Affiliations:** 1 Department of Neuroscience, University of Virginia School of Medicine, Charlottesville, VA USA; 2 Center for Brain Immunology and Glia, University of Virginia School of Medicine, Charlottesville, VA USA

**Keywords:** CSF1R inhibitor, PLX3397, Microglia, LPS, Cytokines, Sickness behavior, Peripheral immune cells

## Abstract

Microglia, the primary immune cells of the central nervous system (CNS), are derived from the yolk sac and populate the brain during development. Once microglia migrate to the CNS, they are self-renewing and require CSF1R signaling for their maintenance. Pexidartinib (PLX3397, PLX), a small molecule inhibitor of the CSF1R, has been shown to effectively deplete microglia since microglial maintenance is CSF1R-dependent. There have, however, been several conflicting reports that have shown the potential off-target effects of PLX on peripheral immune cells particularly those of lymphoid origin. Given this controversy in the use of the PLX family of drugs, it has become important to ascertain to what extent PLX affects the peripheral immune profile in lymphoid (spleen, and bone marrow) and non-lymphoid (kidney, lungs, and heart) organs. PLX3397 chow treatment at 660 mg/kg for 7 days significantly reduced CD45^+^ macrophages, CX3CR1-GFP cells, CD11b^+^CD45^intermediate^ cells, and P2RY12 expression in the brain. However, there were minimal effects on peripheral immune cells from both lymphoid and non-lymphoid organs except in the heart where there was a significant decrease in CD3^+^ cells, inflammatory and patrolling monocytes, and CD11b^+^Ly6G^+^ neutrophils. We then stimulated the immune system with 1 mg/kg of LPS which resulted in a significant reduction in the number of innate immune cells. In this context, PLX did not alter the cytokine profile in the serum and the brain of naïve mice but did so in the LPS-stimulated group resulting in a significant reduction in TNFα, IL-1α, IFN-γ and IL-1β. Furthermore, PLX did not alter locomotor activity in the open field test suggesting that microglia do not contribute to LPS-induced sickness behavior. Our results provide an assessment of immune cell populations with PLX3397 treatment on brain, lymphoid and non-lymphoid organs without and during LPS treatment that can serve as a resource for understanding consequences of such approaches.

**Supplementary Information:**

The online version contains supplementary material available at 10.1186/s12974-023-02924-5.

## Background

Microglia are the primary immune cells of the central nervous system (CNS) and make up 5–10% of the cells in the CNS [40]. Once microglia migrate to the CNS during development, they become self-renewing and require colony-stimulating factor 1 receptor (CSF1R) signaling for their maintenance [17, 19, 27, 53, 73, 76]. CSF1 acting through CSF1R controls the differentiation of macrophages from monocytes when recruited into peripheral tissues. Pexidartinib (PLX3397), a small molecule inhibitor of CSF1R, has been shown to effectively deplete microglia in a dose and duration-dependent manner since microglia renewal and proliferation are CSF1R-dependent [8, 17, 24, 29, 33, 66]. PLX3397 is an oral tyrosine kinase inhibitor that is in phase 1–3 clinical trials for the treatment of different cancers [10]. PLX3397 has also been reported to interfere with other tyrosine kinases c-Kit and Fms-like tyrosine kinase 3 (FLT3) [67], which could have an impact on other immune cells. PLX3397 is also being tested for its potential in treating non-cancerous diseases such as rheumatoid arthritis and multiple sclerosis [52].

There have, however, been several reports of off-target effects of PLX on peripheral immune cells primarily those of the lymphoid origin [29, 43]. Thus, it has become important to ascertain to what extent PLX3397 affects the peripheral immune profile since we recently documented effects of PLX3397 at a high concentration of 660 mg/kg for 7 days on the vasculature [8] and seizures [24]. Here, we examined consequences to the peripheral immune profile in lymphoid (spleen, and bone marrow) and non-lymphoid organs (kidney, lung, and heart) as well as serum and brain cytokines, sickness behavior, and downstream cellular signaling kinases.

We report that PLX3397 did not alter the number of peripheral immune cells from both myeloid and lymphoid origin in lymphoid and non-lymphoid organs except in the heart where we observed a significant reduction in some immune cells. Furthermore, the treatment of mice with 1 mg/kg of LPS resulted in a significant reduction in the numbers of peripheral innate immune cells but LPS-induced sickness behavior was preserved with PLX3397 treatment suggesting a sufficiently intact sickness behavior response without microglia. Finally, PLX3397 did not alter cytokines nor cellular kinases in homeostatic conditions, however, following LPS stimulation, PLX3397 resulted in significant reductions in IFN-γ in serum and IL-1α, IL-1β, and TNFα in the brain. Our results, therefore, suggest that PLX3397 significantly depletes the microglia population in the brain but does not have a dramatic effect on the peripheral immune profile in both lymphoid and non-lymphoid organs (except for innate immune cells following LPS treatment) nor the serum and brain cytokines, and downstream cellular signaling kinases during Gram-negative bacterial infection.

### PLX3397 eliminates microglia in the mouse brain

**Fig. 1:**
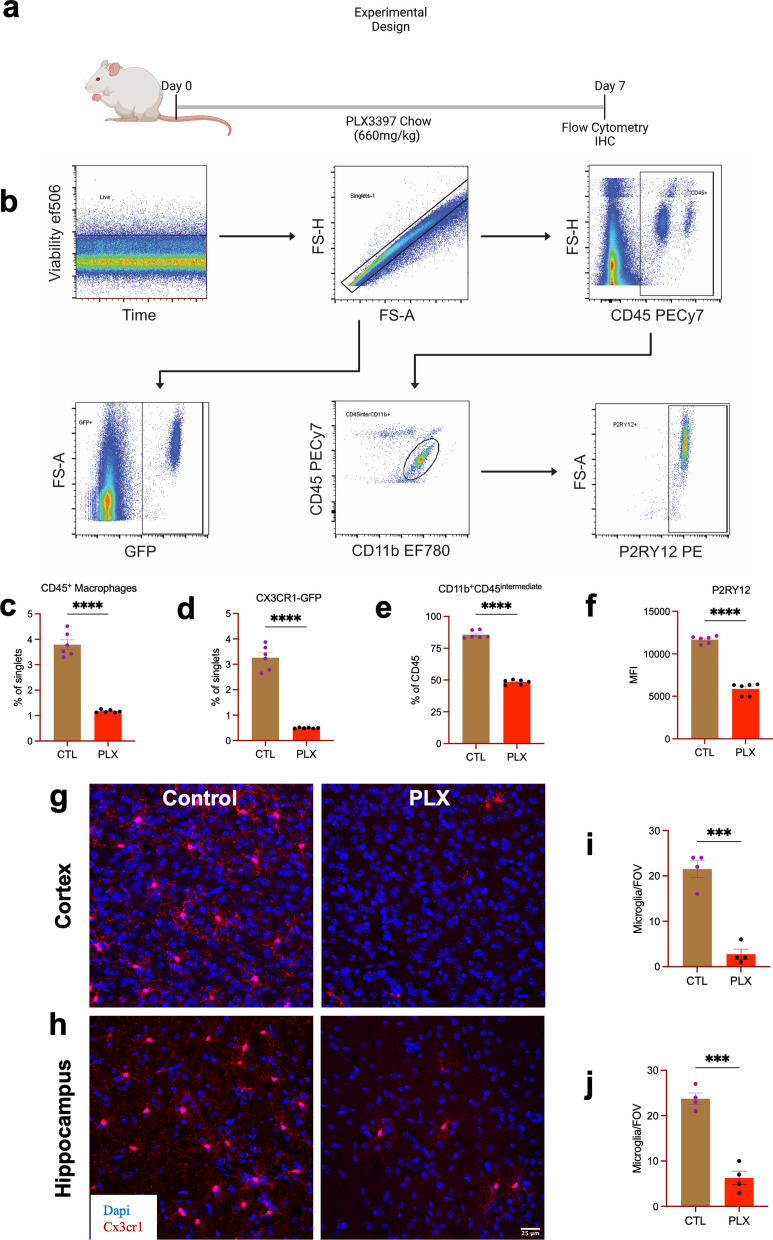
PLX3397 eliminates microglia in the mouse brain. **a** Diagram of experimental illustration of mice placed on PLX3397 for 7 days followed by flow cytometric and immunohistochemistry for microglial density. **b** Representative image showing gating strategy and dot plot of CD45^inter^CD11b^+^. **c** Quantification of CD45^+^ macrophage from single cell population from the brain. **d** Quantification of CX3CR1-GFP + population following PLX3397 chow for seven days. **e** Quantification of the percentage of CD45^inter^CD11b^+^ (microglia) population from CD45^+^ cell. **f** Quantification of P2RY12 expression from CD45^inter^CD11b^+^ population. Representative images of the cortex (**g**) and the hippocampus (**h**) showing microglia after seven days of PLX3397 in chow. **i**, **j** Quantification of microglial density from the cortex (**i**) and the hippocampus (**j**) per field of view. Data were analyzed with unpaired Student’s *T*-test, *n* = 4–6, and data represented by mean ± SEM, ***p < 0.001, ****p < 0.0001

The extent of microglia depletion in the mouse brain was determined using flow cytometry and immunohistochemistry (IHC). We found that mice placed on PLX3397 (660 mg/kg) for 7 days (Fig. 1a) had a significantly reduced number of CD45^+^ macrophages and CX3CR1^GFP/+^ cells in the brain (Fig. 1b, c). We also found that CD11b^+^CD45^intermediate^ (microglia) cells were significantly reduced in the brain compared to mice fed control chow (Fig. 1d, e). The significant reduction in microglial number was also associated with a significant reduction in P2RY12 (a microglial-specific marker) expression in the PLX3397 fed group (Fig. 1f). In addition, IHC for CX3CR1^GFP/+^ showed that microglia were significantly reduced in the cortex and hippocampus compared to mice fed control chow (Fig. 1g–j) resulting in 86.8% and 73.5% decrease in microglia/field of view (FOV) following 7 days of PLX3397 diet in the cortex and hippocampus, respectively. This represents a ~ 80.2% decrease in microglia in the brain by IHC. Taken together, our data show that 7 days of PLX3397 (660 mg/kg) in chow significantly reduced CD45^+^ macrophages, microglial density, and P2RY12 expression in mice indicating an effective depletion of tissue-resident macrophages in the CNS.

### PLX3397 does not affect immune cell numbers in lymphoid organs (bone marrow and spleen)

**Fig. 2:**
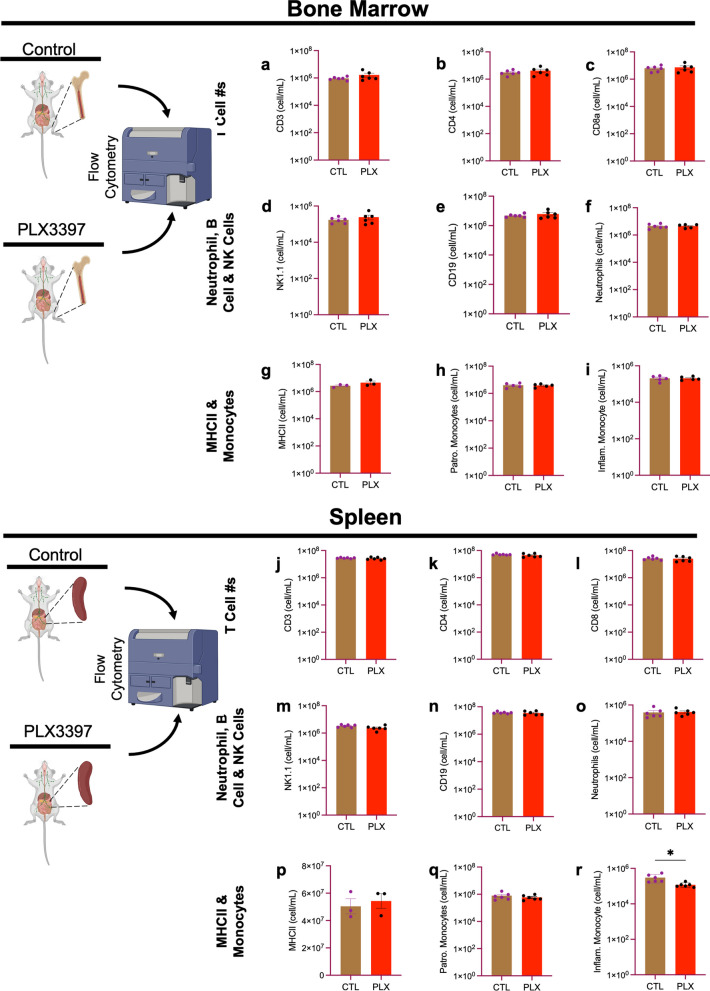
PLX3397 does not affect immune cell numbers in lymphoid and non-lymphoid organs (bone marrow and spleen). Bar graphs show the number of immune cells from the bone marrow: **a** CD3^+^, **b** CD4^+^, **c** CD8a^+^, **d** NK1.1^+^, **e** CD19^+^, **f** CD11b^+^Ly6G^+^, **g** MHCII^+^, **h** CD11b^+^Ly6C_low_ (patrolling monocytes), **i** CD11b^+^Ly6C^high^ (inflammatory monocytes); and the spleen: **j** CD3^+^, **k** CD4^+^, **l** CD8a^+^, **m** NK1.1^+^, **n** CD19^+^, **o** CD11b^+^Ly6G^+^, **p** MHCII^+^, **q** CD11b^+^Ly6C_low_ (patrolling monocytes) , **r** CD11b^+^Ly6C^high^ (inflammatory monocytes). Data were analyzed with unpaired Student’s T-test, n = 3–6, and data represented by mean ± SEM, *p < 0.05

Having confirmed an effective depletion of brain-resident macrophages (microglia) by PLX3397, we sought to interrogate its effects on immune cell numbers in the periphery during the same period. We began by examining prime lymphoid organs. The bone marrow is one of the main lymphoid organs where immature lymphocytes differentiate to mature ones and subsequently migrate to a secondary lymphoid organ like the spleen. Several conflicting data have reported that CSF1R inhibitors impact peripheral immune cell numbers including classical inflammatory monocytes (CD11b^+^Ly6C^hi^) or the non-classical inflammatory monocytes (CD11b^+^Ly6C^low^) [29, 33, 43, 49, 66, 78]. To determine whether a high dose of PLX3397 impacts peripheral myeloid and lymphoid cell numbers in the homeostatic state, we quantified different populations of immune cells in the bone marrow and spleen in mice fed a high dose of PLX3397 for 7 days. We employed the gating strategy shown in Additional file 1: Fig. S1 in determining the various cell populations except in non-lymphoid organs where we had to first gate on CD45 from the size gate. We found that PLX3397 had no effect on the numbers of myeloid and lymphoid cells in the bone marrow and spleen (Fig. 2a–q). However, we found a marginal but significant depletion of Ly6C^low^ monocytes in the spleen of PLX3397-fed mice (Fig. 2r). Our data suggest that a high dose of PLX3397 for 7 days does not have a dramatic effect on peripheral immune cell numbers in the bone marrow and spleen.

### Immune cell numbers in non-lymphoid organs (lungs and kidneys) are not altered by PLX3397

**Fig. 3:**
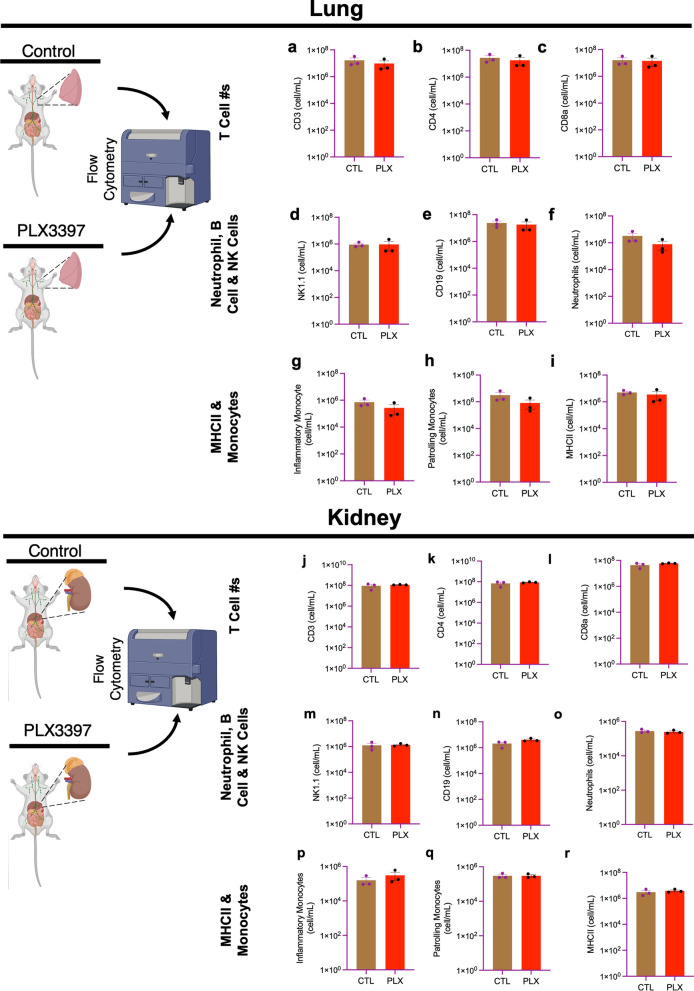
Immune cells in non-lymphoid organs (lung and kidney) are not altered by PLX3397. Bar graphs show the number of immune cells from the lungs: **a** CD3^+^, **b** CD4^+^, **c** CD8a^+^, **d** NK1.1^+^, **e** CD19^+^, **f** CD11b^+^Ly6G^+^, **g** CD11b^+^Ly6C^high^(inflammatory monocytes), **h** CD11b^+^Ly6C_low_ (patrolling monocytes), **i** MHCII^+^; and the kidney: **j** CD3^+^, **k** CD4^+^, **l** CD8a^+^, **m** NK1.1^+^, **n** CD19^+^, **o** CD11b^+^Ly6G^+^, **p** CD11b^+^Ly6C^high^ (inflammatory monocytes), **q** CD11b^+^Ly6C_low_ (patrolling monocytes), **r** MHCII^+^. Data were analyzed with unpaired Student’s *T*-test, *n* = 3 each, and data represented by mean ± SEM

We next evaluated effects of PLX on non-lymphoid organs including the lungs and kidney. We observed that there was no significant effect on the myeloid and lymphoid cell populations in both the lung and kidney (Fig. 3a–r) indicating that a high dose of PLX3397 for 7 days does not negatively impact peripheral immune cell numbers in the lungs and/or kidneys.

### PLX3397 has a minor effect on immune cell numbers in the heart

**Fig. 4:**
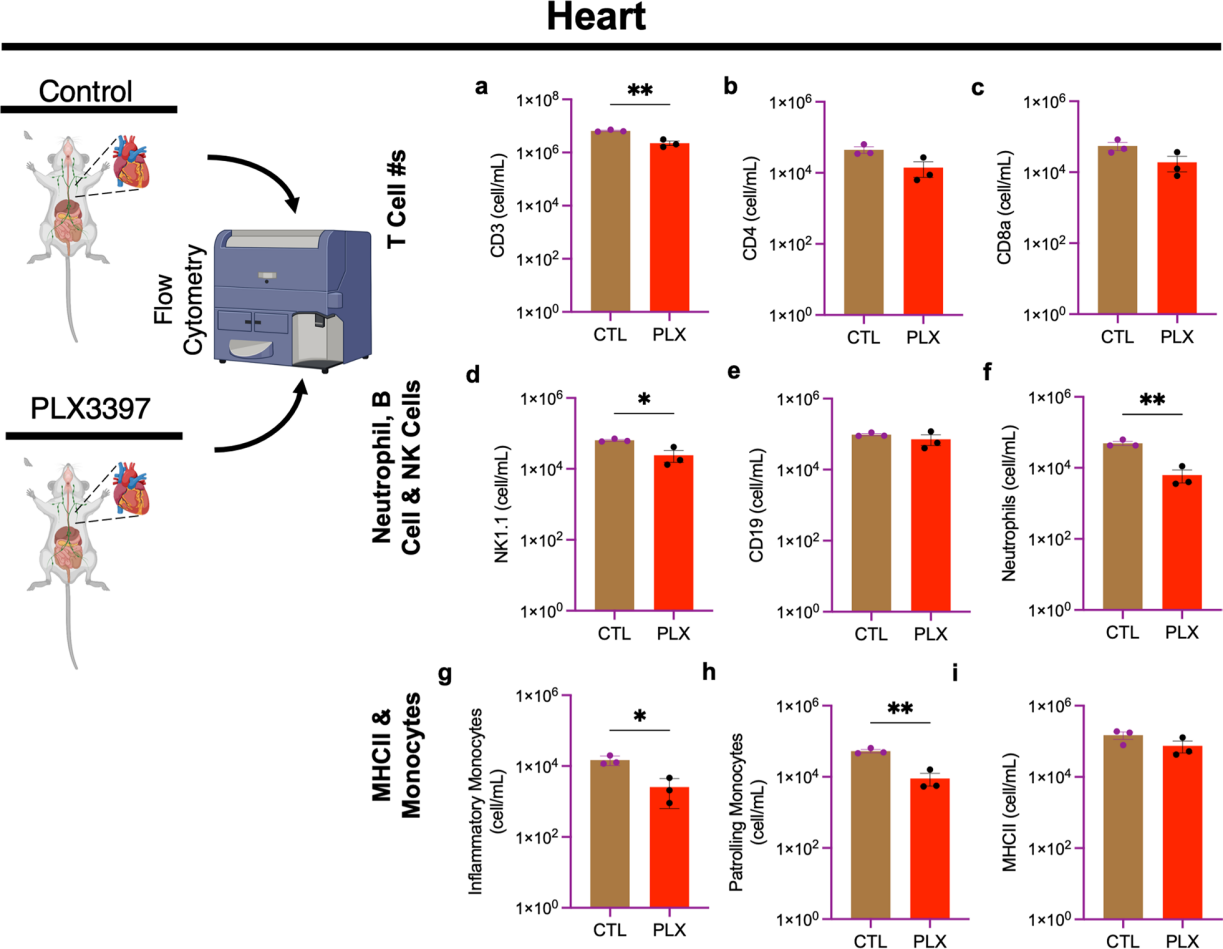
PLX3397 has some minor effect on immune cell numbers in the heart. Bar graphs show the number of immune cells from the heart: **a** CD3^+^, **b** CD4^+^, **c** CD8a^+^, **d** NK1.1^+^, **e** CD19^+^, **f** CD11b^+^Ly6G^+^ (neutrophils), **g** CD11b^+^Ly6C^high^ (inflammatory monocytes), **h** CD11b^+^Ly6C_low_ (patrolling monocytes), **i** MHCII^+^. Data were analyzed with unpaired Student’s *T*-test, *n* = 3, and data represented by mean ± SEM, *p < 0.05, **p < 0.01

In addition to the kidney and lung, we also examined the heart as a non-lymphoid organ. Contrary to results obtained for the lung and kidney, we observed that a high dose of PLX3397 for 7 days resulted in a significant decrease in CD3^+^ (Fig. 4a), NK1.1^+^ (Fig. 4d), CD11b^+^Ly6G^+^ (Fig. 4f), Ly6C^hi^, and Ly6C^low^ (Fig. 4g, h) cells compared to control. However, there was no significant difference between PLX3397 and the control chow-fed group with CD4^+^, CD8a^+^, CD19^+^, and MHC II^+^ cells (Fig. 4b, c, e, i). Taken together, these data show that PLX3397 causes a reduction in some peripheral immune cell populations in the heart.

### Innate immune cell numbers are negatively impacted by PLX3397 in lymphoid and non-lymphoid organs after LPS treatment

**Fig. 5:**
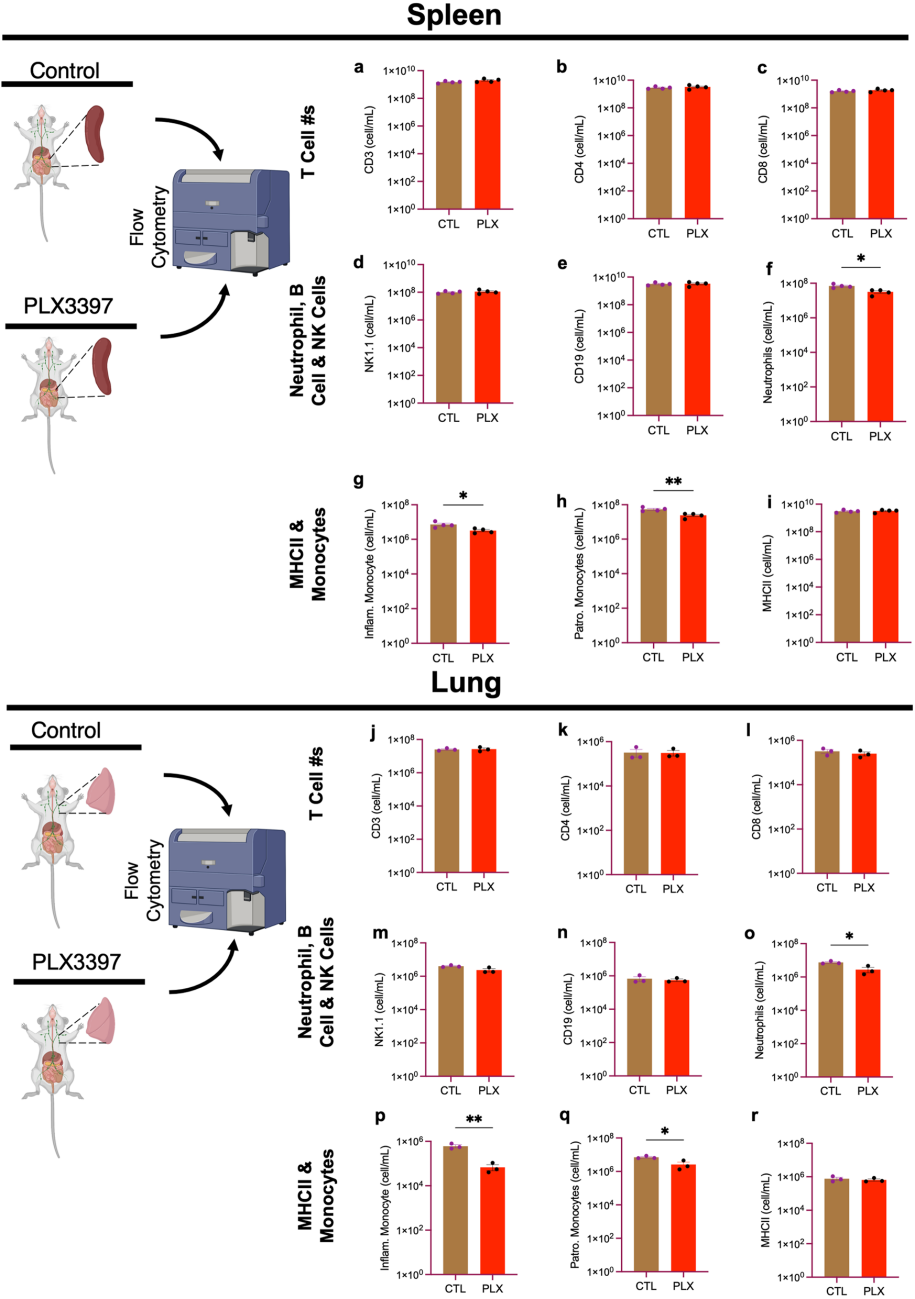
Innate immune cell numbers are negatively impacted by PLX3397 in lymphoid and non-lymphoid organs after LPS infection. Bar graphs show the number of immune cells from the spleen: **a** CD3^+^, **b** CD4^+^, **c** CD8a^+^, **d** NK1.1^+^, **e** CD19^+^, **f** CD11b^+^Ly6G^+^ (neutrophils), **g** CD11b^+^Ly6C^high^ (inflammatory monocytes), **h** CD11b^+^Ly6C_low_ (patrolling monocytes), **i** MHCII^+^; and the lung: **j** CD3^+^, **k** CD4^+^, **l** CD8a^+^, **m** NK1.1^+^, **n** CD19^+^, **o** CD11b^+^Ly6G^+^ (neutrophils), **p** CD11b^+^Ly6C^high^ (inflammatory monocytes), **q** CD11b^+^Ly6C_low_ (patrolling monocytes), **r** MHCII^+^. Data were analyzed with unpaired Student’s *T*-test, *n* = 3–4, and data represented by mean ± SEM, *p < 0.05, **p < 0.01

Next, we attempted to determine the impact of PLX3397 on immune cell numbers during a Gram-negative bacterial infection. Lipopolysaccharide (LPS), which is a component of the Gram-negative bacteria, is a commonly used endotoxin that serves as a murine model to induce systemic and neuroinflammation and mimic Gram-negative bacterial infection. To determine to what extent PLX3397 impacts immune cell numbers from both myeloid and lymphoid compartments using the spleen (lymphoid organ) and lung (non-lymphoid organ), we stimulated the immune system with 1 mg/kg of LPS on the 7th day of a high PLX3397 diet then performed flow cytometry evaluation. Our results show that there was no significant effect on the number of adaptive immune cells (CD3^+^, CD4^+^, CD8^+^, NK1.1^+^, and CD19^+^) and MHCII^+^ cells in both the spleen and lung at 6 h. However, we observed a significant decrease in innate immune cell numbers including CD45^+^CD11b^+^Ly6C^hi^ (inflammatory) monocytes, CD45^+^CD11b^+^Ly6C^low^ (patrolling) monocytes, and CD11b^+^Ly6G^+^ neutrophils in the PLX-fed group compared to the control group in both the spleen and the lung (Fig. 5a–r). Taken together, PLX3397 treatment yields a significant decrease in cells involved in the innate immune system following LPS stimulation.

### PLX3397 alters inflammatory cytokine levels (but not cellular kinases) in the serum and brain following LPS treatment

**Fig. 6:**
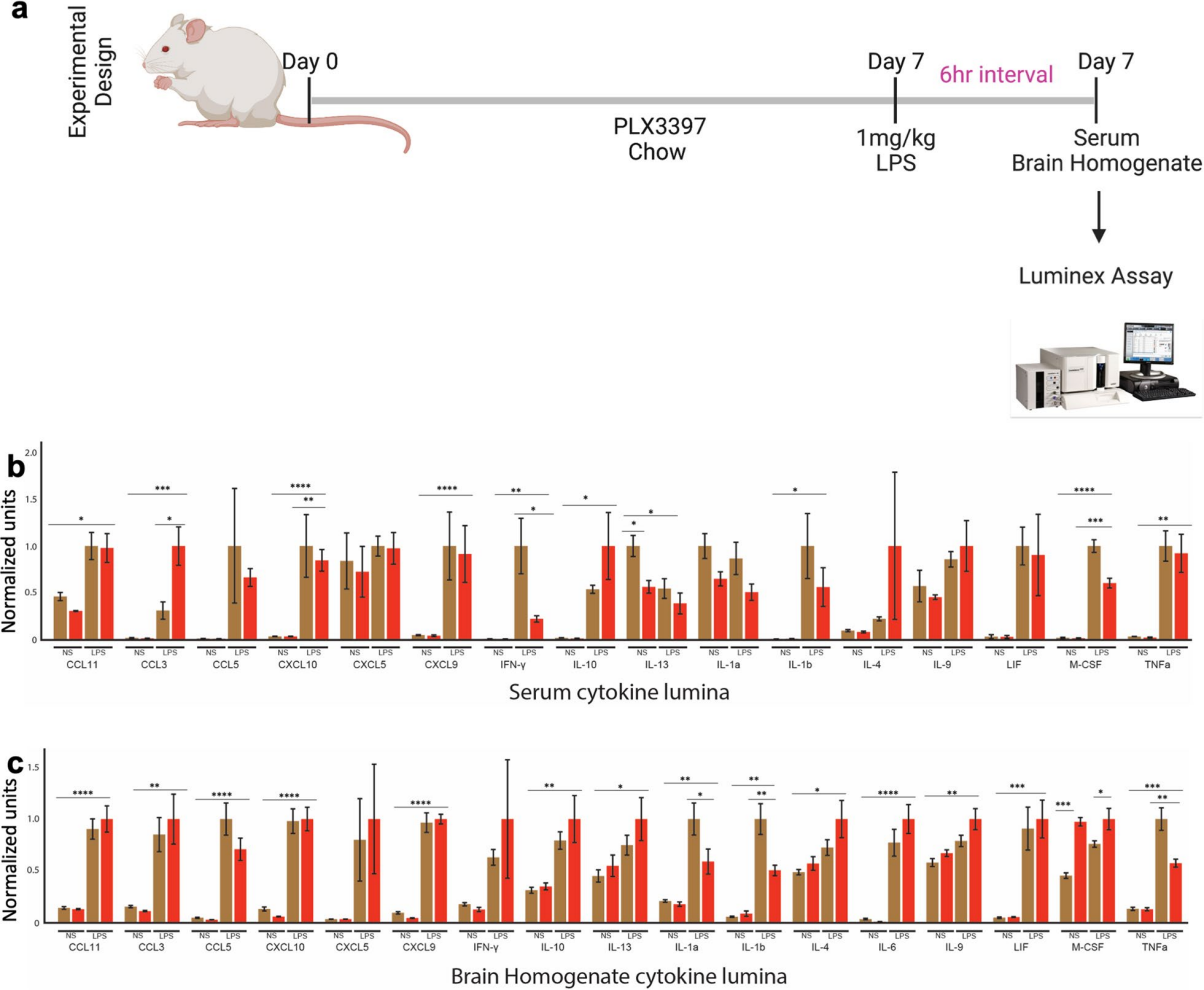
PLX3397 decreases inflammatory cytokines in serum and brain homogenate following LPS. **a** Illustration of experimental design showing mice placed on PLX3397 for seven days followed by Luminex assay for serum and brain homogenate cytokine profiling. **b** serum cytokine profile **c** brain homogenate cytokine profile. Data were analyzed with unpaired Student’s *T*-test, *n* = 4–5, and data represented by mean ± SEM, *p < 0.05, **p < 0.01.***p < 0.001, ****p < 0.0001

Following the observation that LPS treatment results in a decrease in innate immune cell numbers, we decided to ascertain to what extent PLX impacts cytokine secretion following endotoxin stimulation using 1 mg/kg of LPS. Following this treatment, we performed a Luminex assay assessment after 6 h on serum (Fig. 6a). Our results show that in the basal state, there was no significant difference between control and PLX3397-fed mice in all the serum cytokines profiled except for IL-13 which was significantly reduced in the PLX3397-fed group compared to control (Fig. 6b). However, stimulation with LPS resulted in a significant decrease in M-CSF, IFNγ, and CXCL10 in the PLX3397-fed group, while there was a significant increase in CCL3 in the control group (Fig. 6b). In addition, except for M-CSF which was significantly increased in the PLX3397-fed group, all profiled brain cytokines were not altered in the homeostatic state with the high dose of PLX3379 for 7 days (Fig. 6c). Consequently, following LPS stimulation, we observed a significant decrease in TNFα, IL-1α, and IL-1β in the PLX3397-fed group; and an increase in M-CSF in the PLX3397-fed group (Fig. 6c).

We also ascertained to what extent PLX impacts cytokine secretion at 24 h after of LPS stimulation using Luminex assay (Additional file 2: Fig. S2a). Our results show that there was no significant difference between control and PLX3397-fed mice in the anti-inflammatory profile (IL-4, IL-13), except for IL-10 which was significantly reduced in PLX3397-fed mice compared to control (Additional file 2: Fig. S2b). We also observed a non-significant effect in the main pro-inflammatory profiles (IL-1α, IL-1β, TNFα, and IFN-γ) we examined. However, there was a significant increase in IL-6 in LPS-treated group compared to the normal saline group (Additional file 2: Fig. S2c). In addition, there was no significant difference in IL-6 secretion following LPS stimulation in both control and PLX3397-fed mice (Additional file 2: Fig. S2c). We observed that chemokine (CCL11, CXCL2, CXCL1, CCL5) secretion was not significantly impacted by PLX3397 following LPS stimulation. However, MIG (CXCL9) secretion was significantly decreased in PLX3397-fed mice following LPS stimulation compared to the control chow-fed mice (Additional file 2: Fig. S2d).

We next examined the effect of a high dose of PLX3397 on serum and brain downstream cellular signaling pathways. Our data show that PLX3397 alone or in combination with LPS did not significantly alter the total levels of any of the cellular (CREB, JNK, NF-kB, p38, ERK1/2, Akt, p70S6K, STAT3, STAT5) pathways we examined in both serum and brain (Additional file 3: Fig S3a, b). Our findings are congruent with previous data that reported that 50 mg/kg of PLX3397 every second day for three weeks does not alter the cellular production of pJNK and pERK1/2 in the visceral fat [49]. In all, our data show that CSF1R inhibition using PLX3397 does not impact peripheral immune cells’ ability to secrete interleukins and chemokines both in the basal state and during Gram-negative bacterial infection.

### PLX3977 does not block the sickness-inducing effects of LPS treatment

**Fig. 7:**
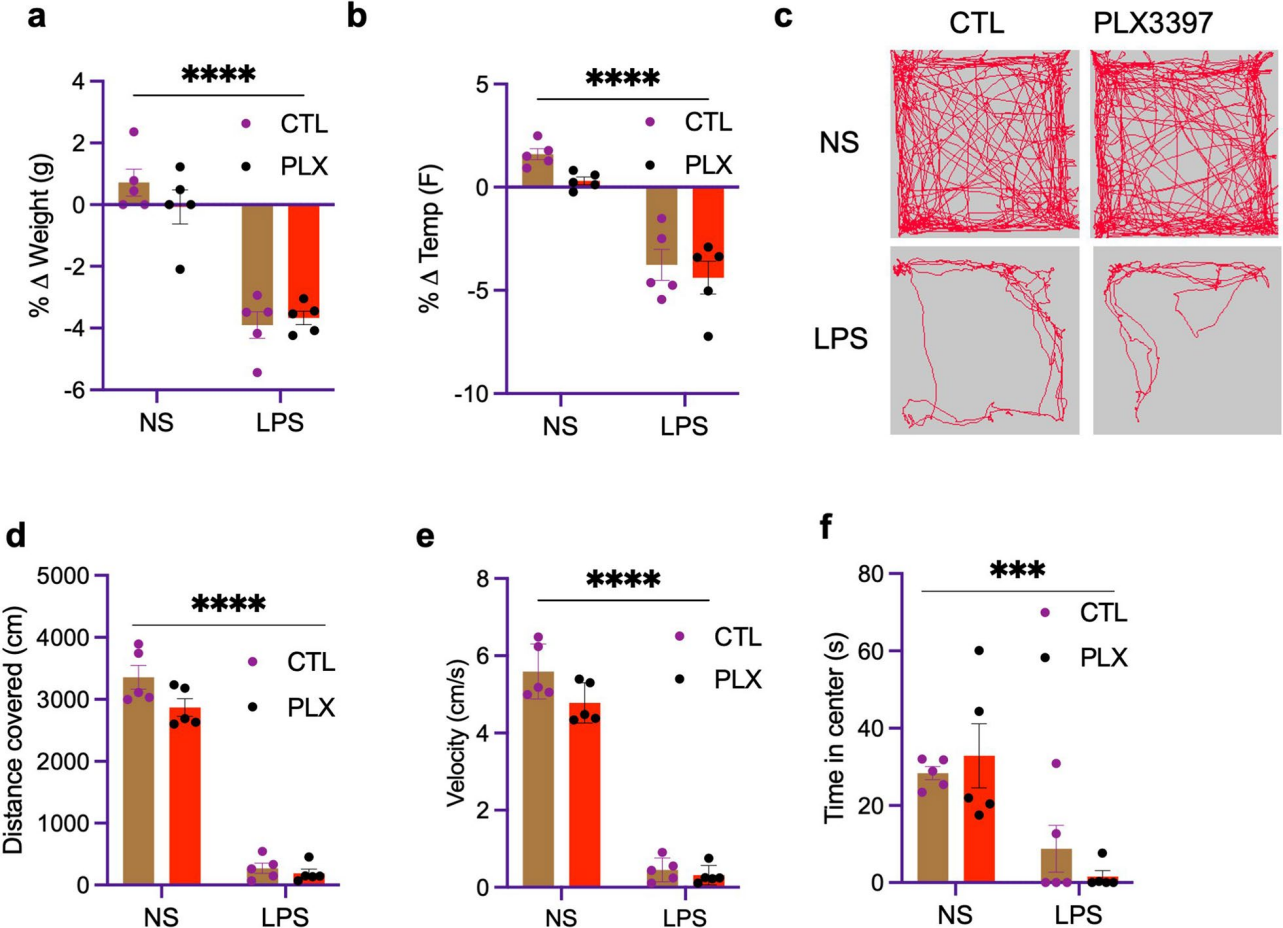
PLX3397 does not affect LPS-induced sickness behavior. **a**, **b** Effects of LPS on **a** body weight and **b** body temperature measured 6 h after intraperitoneal LPS injection in PLX3397-fed mice compared to control chow-fed mice, **c** representative images of mouse exploration without and following LPS. **d–f** Quantification of locomotor activity measured by **d** distance traveled, **e** velocity and **f** time in center in an open-field test carried out 6 h post-LPS. Data represented by mean ± SEM, *n* = 5, ***p < 0.001, ****p < 0.0001

Given the evidence above that PLX3397 significantly depleted microglia and altered the expression of some inflammatory cytokines in an LPS-treatment context, we sought to investigate the possible roles of microglia in sickness behavior following LPS stimulation since the mice presented with sickness determinants despite the absence of microglia. Measurement of body weight and temperature are typically used to monitor the health or effects of anaphylactic symptoms in animals as one of the several determinants of sickness [22, 35, 41]. Our data show that a high dose of PLX3397 does not alter the body weight (Fig. 7a) and temperature (Fig. 7b). However, following LPS treatment both PLX3397-fed and control groups had a significant drop in body weight and temperature within 6 h compared to the normal saline-treated group (Fig. 7a, b). Several studies have shown that LPS in animals results in sickness behavior which is characterized by but not limited to decreased locomotor activity and appetite [7, 30, 36]. In the open field, the total distance traveled is often used as a measurement of sickness behavior in an inflammatory experimental paradigm. Our data show that PLX3397 at a high dose does not alter exploratory behavior in the open field (Fig. 7g). However, LPS treatment at 6 h significantly reduced the distance traveled (Fig. 7e), the movement velocity (Fig. 7f), and time in the center (Fig. 7g) compared to the normal saline-treated control group. Our data, therefore, suggest that in the absence of microglia, other cells are sufficient to mediate sickness behavior following LPS treatment.

## Discussion

Microglia are the main innate immune cell in the CNS and have been implicated as a driving mechanism in several diseases of the nervous system. One approach to interrogate microglial roles in the CNS is to examine the consequence of microglial elimination on various aspects of CNS structure or function. Earlier techniques to eliminate microglia did so either *transiently*, e.g., by local administration of clodronate [38, 54, 69, 74], *pharmacogenetically* using diphtheria toxin or similar strategies [50, 58, 79] or *genetically* targeting genes for myeloid cell survival with developmental consequences as well as effects on other myeloid cells [5, 12, 25, 48, 60]. Moreover, some of these techniques such as the pharmacogenetic ones have since been discovered to elicit indirect additional effects than microglial elimination in the brain. These include for example, physical alterations to the brain ventricles, increased glial reactivity, increased cytokine levels and some dysfunctional motor behaviors [4, 9, 59]. Therefore, these techniques suffer from elements of insufficient temporal control or unintended confounds in addition to eliminating microglia.

In 2014, with the introduction of the PLX family of CSF1R inhibitors as an alternative for microglial elimination, some of these concerns were mitigated by the approach to treat mice with these drugs through the chow feed which could allow for (1) temporal control (treatment could be commenced and terminated at will for short or long durations as needed), without (2) the known confounds of other approaches such as alterations to brain ventricle size, increased cytokine levels, astroglial activation and motor dysfunction [4, 9, 59]. However, recent studies suggests that PLX treatment has effects on the peripheral immune system including with cells outside the myeloid lineage such as B cells and T cells [43] raising a need to further characterize the effect of the PLX family of CSF1R antagonists. Here, we focused on the effects of PLX3397 rather than that for PLX5622 for four reasons. First, PLX3397 is more *widely used* in the literature (a PubMed search yields 293 results versus 170 for PLX5622). Second and possibly explaining the first reason, PLX3397 is *more widely accessible* to researchers because it is less expensive compared to PLX5622. Third, PLX3397 is used in more clinical trials (24 with several in 2020 and at least one in 2021 on the clinicaltrials.gov website) than PLX5622 (only 2 and the last was in 2015) requiring a better understanding of its effects with its *greater clinical use*. Fourth, for basic science research, we were looking for an approach that *rapidly* eliminates microglia at low concentrations. The widely used concentration for PLX3397 is already high at 1200 mg/kg and that for PLX3397 was 290 mg/kg which we could increase to the maximum that has been used in the literature at 660 mg/kg. Thus, for experimental, financial, clinical, and practical reasons, we choose to conduct the study with PLX3397 though a study of PLX5622’s effects would be just as good and is much needed. At this dose, we demonstrated effective microglial elimination within a relatively short duration of 7 days (Fig. 1). We selected this dose to provide a *rapid* and *effective* depletion of microglia which we have used and confirmed in other previous from our lab [8, 24]. While lower doses could eliminate microglia by 2–3 weeks, we wanted a *rapid* elimination paradigm because some results from previous studies such as [44] have shown effects on the periphery which could result from a longer exposure to the diet. However, we had not previously examined the effects of this treatment regimen outside the brain.

We report that this regimen did not appreciably alter the peripheral immune cell profile in both lymphoid (Fig. 2) and non-lymphoid (Figs. 3, 4) organs, and cytokine profile in serum and brain in the basal state (Fig. 6). However, following LPS stimulation, we observed that innate immune cells from lymphoid and non-lymphoid organs were significantly decreased in the PLX3397-fed group (Fig. 5). In addition, we observed that PLX treatment coupled with LPS stimulation decreased the secretion of IFN-γ in serum and TNFα, IL-1α, and IL-1β in the brain (Fig. 6). However, these significantly decreased secretions of inflammatory cytokines did not result in the alterations of downstream total protein levels of cellular signaling pathways (Additional file 2: Fig. S2). Furthermore, we demonstrated that pharmacological microglial depletion using PLX3397 did not abrogate sickness behavior in mice following LPS stimulation (Fig. 7), indicating that the peripheral immune system remains intact in the absence of microglia to mediate sickness behavior. Similar findings have recently been reported using PLX5622 in mice and microglial depletion induced by the administration of diphtheria toxin to Cx3cr1-Dtr transgenic rats [71]. Taken together, these findings suggest that PLX3397 at a dose of 660 mg/kg eliminates microglia by 7 days with little impact on peripheral immune cell numbers in the naïve state but limits the production of some innate immune cells in the periphery, and cytokine storm during a Gram-negative bacterial infection. Finally, we show that microglia are not required for sickness behavior. It is worth noting that our studies leave open the possibility that the results found in the periphery following PLX3397 treatment to eliminate myeloid cells may result indirectly from consequences of microglial depletion or directly from PLX3397 effects in the periphery.

Microglial survival, maintenance, and proliferation are essentially dependent on CSF-1 and its receptor CSF1R. CSF1R is also expressed on peripheral immune cells specifically macrophages and monocytes. Elmore et al. [17] reported that PLX3397 at a dose of 290 mg/kg exerted a limited influence on other immune cells in the periphery. In addition, several other studies have reported inconsistency on the effect of PLX3397 on peripheral immune cells. Merry et al. [49] reported that PLX3397 (50 mg/kg) for 21 days through oral gavage significantly decreased the numbers of macrophages in the adipose tissue, but not of circulating myeloid cells. Szalay et al. [66] reported that PLX3397 (290 mg/kg) in feed for 21 days did not alter monocyte/granulocyte and lymphocyte populations in both blood and spleen. Other studies have also indicated that PLX3397 results in a significant reduction in red blood cells, hemoglobin, platelets, dendritic cells, Ly6C^−^ monocytes [61], and splenic red pulp macrophages [29]. A plausible interpretation of these results may be due to the different concentrations, duration, and routes of administration of PLX3397 treatment and possible differences in vivarium used in these studies.

In addition, PLX5622, a more specific CSF1R inhibitor has also been shown to significantly deplete macrophages and monocytes in both the circulation and the liver [75]. Another study using PLX5622 suggested it is not CNS-specific, with long-term effects on the myeloid and lymphoid compartments of bone marrow, spleen, and blood [43]. In contrast, our results show that the numbers of immune cells from both myeloid and lymphoid compartments were not impacted by PLX3397 at a concentration of 660 mg/kg for 7 days. However, upon stimulation with LPS, we observed a significant decrease in immune cells of the myeloid compartment specifically neutrophils, classical and non-classical monocytes in both lymphoid and non-lymphoid organs. These results suggest that each study using these drugs should specifically analyze the peripheral immune compartment to clarify effects from the route of delivery, duration of treatment and concentration of drug used.

Some limitations of the current study are noteworthy. First, while we did not find significant effects of PLX3397 treatment on peripheral immune cells, microglia are not the only myeloid cells eliminated with a PLX3397 treatment. Indeed, border associated (meningeal and perivascular) macrophages are also eliminated with PLX3397 treatment (data not shown) requiring caution in assuming microglial specificity among brain macrophages when using PLX3397. Second, our study was strictly limited to the depletion paradigm, though there is an established paradigm of repopulation that ensues upon PLX3397 drug withdrawal that could also have impacts on brain and peripheral immune cell density that would be worth investigating in future since it is has now been show in several paradigms that following repopulation in aged mice, improvements in neuronal function [18] and recovery from pathology [29, 45, 62] are evident. Whether these can be a result of changes to peripheral cell populations, however, has not been investigated in those paradigms nor in our paradigm. Third, our analysis was done on groups of 3–6 mice and we acknowledge that our study would be improved by increasing the number of mice per group. Fourth, we used a specific LPS-treatment paradigm of a single dose at 1 mg/kg and examination at a single timepoint at 24 h. We report our findings here but also acknowledge that other LPS treatment concentrations and timepoints may yield different results. Finally, while we show limited effects on peripheral immune cell density during PLX3397 treatment, we did not assess the function of these immune populations and thus cannot rule out functional consequences to these cell populations independent of effects on density. All these limitations are of course of interest for future studies.

In conclusion, in the current study, we report a brain-selective effect of a 7-day 660 mg/kg PLX3397 treatment on the myeloid cell population when compared to peripheral lymphoid (bone marrow and spleen) and non-lymphoid (kidney, lung, and heart) organs. PLX3397 at this concentration and for this duration had minimal effects on various immune cell populations in these organs in the naïve state. However, following LPS-induced infection, PLX3397 treatment at this concentration and duration resulted in a reduction in the number of some immune cell populations in the periphery. These results provide a paradigm that could be of use for further studies in which PLX3397 is used.

## Materials and methods

### Animals

All animal experimentations were carried out in accordance with the relevant guidelines and regulations of the University of Virginia and approved by the Institutional Animal Care and Use Committee with protocol number 4237. The animals were housed under controlled temperature, humidity, and light (12:12 h. light: dark cycle), with food and water readily available ad libitum. Only male mice were used for this study on a C57BL/6J background age 11–13 weeks and consisted of the following genotypes: CX3CR1^GFP/+^ expressing GFP under control of the fractalkine receptor (CX3CR1) promoter [34] and C57Bl/6J mice as wildtype mice. Mice were housed in groups for the experiment without special environmental enrichment.

### Pharmacological elimination of microglia

Microglia were depleted using a potent CSF1R inhibitor, PLX3397, that has been previously shown to lack inflammatory consequences during the elimination process. Microglia from the adult brain were depleted by feeding adult mice with chow containing PLX3397 (660 mg/kg) in the experimental group and control chow containing 75 mg/kg PLX3397 for 7 days. We recently showed that at 75 mg/kg, PLX3397 did not affect microglial numbers in the brain [24].

### Open field behavioral test

The open field test was carried out in a custom-built arena using a white plastic material with 35 (L) × 35 (B) × 21 (H) cm dimensions. Mouse cages were moved into the testing room and allowed to acclimate for 1 h. Illumination in the room was maintained at 150 Lux intensity, temperature, and relative humidity were also relatively constant at 70.2 ± 0.9 °F and 40.9 ± 5%, respectively. All experiments were done during the light cycle. Five mice from the same cage were simultaneously placed into different arenas that had been cleaned with 70% ethanol. Each mouse was placed adjacent to the wall of the arena and allowed to freely explore the space. Their open field activities (horizontal locomotion and mobility) in the arenas were video monitored for 10 min using EthoVision^®^ XT (Noldus, Wageningen, The Netherlands), and then subsequently tracked offline for activity analysis using the same software.

### Lipopolysaccharide administration

To evaluate the effect of LPS on the immune profile, mice placed on a high dose of PLX3379 for 7 days were weighed using an electronic scale (CGOLDENWALL) before and 6 h after i.p injection of 1 mg/kg/bw of LPS prepared from *Escherichia coli* (026:B6, Sigma-Aldrich, St. Louis, USA). Body temperature was also recorded before and after LPS injection using a hand-held medical infra-red thermometer (Model: HG01, China) as previously described [35].

### Cytokine and cell signaling assay

Following 6 h of LPS injections, mice were subjected to cardiac puncture. The blood collected was allowed to coagulate for 30 min at room temperature and subsequently centrifuged for 10 min at 1000×*g*. The supernatant (serum) was collected and stored at – 80 ºC until ready for the Luminex assay. Spleen and lungs were also collected and prepared for flow cytometric staining to profile peripheral immune cells. A different cohort of mice was perfused with ice-cold 1X PBS, the brain was quickly removed and homogenized. The supernatant was collected and stored at – 80 ºC until ready for Luminex assay. Milliplex mouse cytokine/chemokine magnetic bead analysis kit was obtained from Millipore Sigma (MA, USA). For cellular kinases, Milliplex multi-pathway 9-plex magnetic bead kit (48-680MG) was used. Assays were run in duplicate according to the manufacturer’s protocol. Data were collected using the Luminex Intelliflex (Luminex, Austin, TX, USA). Data analysis was performed using the Milliplex Analyst 5.1 software (MA, USA). The multi-pathway 9-plex data were normalized to GAPDH.

### Tissue preparation and immunostaining

For confocal microscopy studies, mice were euthanized with CO_2_ and transcardially perfused with sodium phosphate buffer (PBS; 50 mM at pH 7.4) followed by 4% paraformaldehyde (PFA). All perfusion solutions were chilled on ice prior to use. We used a perfusion pump (Masterflex^®^ Ismatec^®^) at a perfusion flow rate of 7 mL/min. Brains were then fixed in 4% PFA overnight. Using a vibratome (Leica VT100S), 40-μm-thick sections of the brain were cut in chilled PBS. Slices were then stored in cryoprotectant (40% PBS, 30% ethylene glycol, and 30% glycerol) at − 20 °C while further processing took place. Brain sections containing the ventral hippocampus CA1 (Bregma − 3.27 and − 4.03), the frontal cortex (Bregma 2.93 and − 2.57), and the sensorimotor cortex (Bregma − 2.5 and + 2.0) were examined.

### Fluorescence microscopy

Fluorescently immunolabelled brain sections were imaged with a Leica SP8 Laser Confocal Microscope. Image analysis to quantify microglia cell numbers through *z*-stacks was done using ImageJ. Microglia cell number was automatically counted using the ImageJ cell counter plugin.

### Tissue processing and flow cytometry staining

Mice were euthanized with CO_2_ and sprayed with 70% ethanol. The spleen was collected and placed in cold complete RPMI media (cRPMI) (10% FBS, 1% sodium pyruvate, 1% non-essential amino acids, 1% penicillin/streptomycin, 0.1% 2-ME). The spleen was mashed through a 40-μm filter in 50 mL conical using a syringe plunger and wash through with 15-mL cRPMI. The suspension was centrifuged at 1600 rpm for 5 min at 4 °C using Eppendorf Centrifuge 5804R with an S-4–72 rotor. The spleen was resuspended in 2 mL RBC lysis buffer for 2 min and the reaction was stopped by adding 13 mL cRPMI. The suspension was once again centrifuged, and the resulting pellet was resuspended in 5 mL cRPMI and kept on ice.

For the preparation of a single-cell suspension for the bone marrow, the femur and tibia were carefully removed without splintering them to obtain the bone marrow. The bones were placed in a petri dish containing 70% ethanol for 1–2 min to disinfect and transferred to a petri dish containing 4 mL cRPMI on ice. The femora and tibiae were flushed with 10 mL ice-cold cRPMI using a 21G needle attached to a 10-mL syringe. A single-cell suspension was generated by gently triturating the cells through the needle until large clumps were no longer present and the suspension was run through a 40 μm filter in a 50 mL conical tube. The suspension was centrifuged at 1600 rpm for 5 min at 4 °C. The bone marrow was resuspended in 2 mL RBC lysis buffer for 2 min and the reaction was stopped by adding 13 mL cRPMI. The suspension was centrifuged at 1600 rpm for 5 min at 4 °C, the supernatant was aspirated, and the pellet was resuspended in 5 mL cRPMI.

For the preparation of single cells from the kidney, an established protocol was used [47]. Briefly, following mice euthanizer with CO_2_, they were exsanguinated. Both kidneys were removed after careful renal pedicle dissection and decapsulated. Kidneys were finely minced and incubated in collagenase D (2 mg/mL, Sigma-Aldrich, St. Louis, MO, USA) for 30 min at 37 ºC. Single-cell of the kidney was prepared by straining through a 70 μm strainer (Corning).

For the preparation of single-cell suspension from the heart and lung, we adopted previously published protocols [11, 23, 26, 42, 80]. Following euthanizer with CO_2_ and systemic perfusion with ice cold 1 × PBS, organs of choice were carefully dissected and cut into 1-2 mm pieces and incubated for 30 min in cRPMI containing 2 mg/mL collagenase D at 37 ºC. Digested tissue was strained using 70 μm strainer. After washing with cRPMI, cell suspension was then run at room temperature with isotonic Percoll density (GE Healthcare, Chicago, IL, USA) centrifugation (1500×*g* for 30 min in brake-off mode). Collected cells from each organ were washed and resuspended on cRPMI.

Following the generation of a single-cell suspension, cells were counted using automated cell counter (C100, RWD, China), 150 μL (~ 1 × 10E6) of each sample were placed in a 96-well plate and incubated for 10 min in 50 μL Fc block (1:1000, CD16/32, Clone 93, eBioscience) at room temperature. Cells were then incubated in primary antibodies at a concentration of 1:200 and fixable viability dye eFluor 506 (eBioscience) at a concentration of 1:800 for 30 min at 4 °C. Antibody clones used for experiments included: NK1.1 (PK136), CD3e (145–2C11), CD8a (53–6.7), CD4 (RM4–5), CD19 (eBio 1D3), Ly6C (HK1.4), F4/80 (BM8), CD11b (M1/70), Ly6G (1A8), CD45 (30-F11), MHC II (M5/114.15.2), and CD11c (N418) (Invitrogen). After staining, cells were washed twice and fixed overnight in 2% PFA at 4 °C. Cells were washed twice with cell staining buffer (BioLegend, Cat. # 420201) and transferred into a 5 mL Polystyrene round-bottom tube with cell-strainer cap tubes (Falcon), then were analyzed on a Gallios flow cytometer (Beckman-Coulter) by gating on 250,000 live events. Flow cytometry data were analyzed using FlowJo (version 10.8.1).

### Statistical analysis

Data were initially measured for normality and homoscedasticity and upon comparing normal distributions and variances further analyzed with the respective tests. Student’s t-test was used to compare two groups. Other comparisons were evaluated using two-way ANOVA (experiments with 2 variables), followed by Tukey post hoc test for multiple comparisons within the tested groups.

### Supplementary Information

**Additional file 1: Fig S1: **Gating strategy employed in peripheral immune cell population profiling. C57BL6/J mice were fed a chow diet for 7 days containing CSF1R inhibitor PLX3397 (660 mg/kg). Samples were labeled with mixtures of specific antibodies against immune cell populations and subjected to flow cytometric analysis. Singlets were gated FS-A/FS-H, live mononuclear cells were gated based on SS-A/fixable Viability Dye (eFlour 506), then size gated based on SS-A/FS-A to exclude red blood cells. As indicated in the illustration above, a specific population of choice based on the antibody was identified from the size gate.

**Additional file 2: Figure S2.** Seven days of CSF1R inhibition with PLX3397 does not alter serum cytokine/chemokine profile (except IL-10 and CXCL9) 24 h after LPS stimulation. **a.** Illustration of experimental design showing mice placed on PLX3397 for seven days followed by serum Luminex assay for cytokine profiling. **b**. serum cytokine anti-inflammatory profile **c**. serum cytokine pro-inflammatory profile **d**. serum chemokine profile. Data were analyzed with unpaired Student’s T-test, n = 3–5, and data represented by mean ± SEM, *p < 0.05, **p < 0.01.***p < 0.001, ****p < 0.0001.

**Additional file 3: Figure S3. **PLX3397 does not affect cellular kinases in serum and brain homogenate following LPS infection.** a**. serum cellular kinases pathways **b**. brain homogenates cellular kinase pathways. Data were analyzed with Student’s unpaired T-tests, n = 5, and data represented by mean ± SEM.

## Data Availability

All datasets used and/or analyzed in this present study are available from the corresponding author upon request.
