# Supplementary figures and images for: Distinguishing the effects of systemic CSF1R inhibition by PLX3397 on microglia and peripheral immune cells
Source: J Neuroinflammation. 2023 Oct 21;20:242. doi: 10.1186/s12974-023-02924-5 (PMC10590528; doi:10.1186/s12974-023-02924-5)

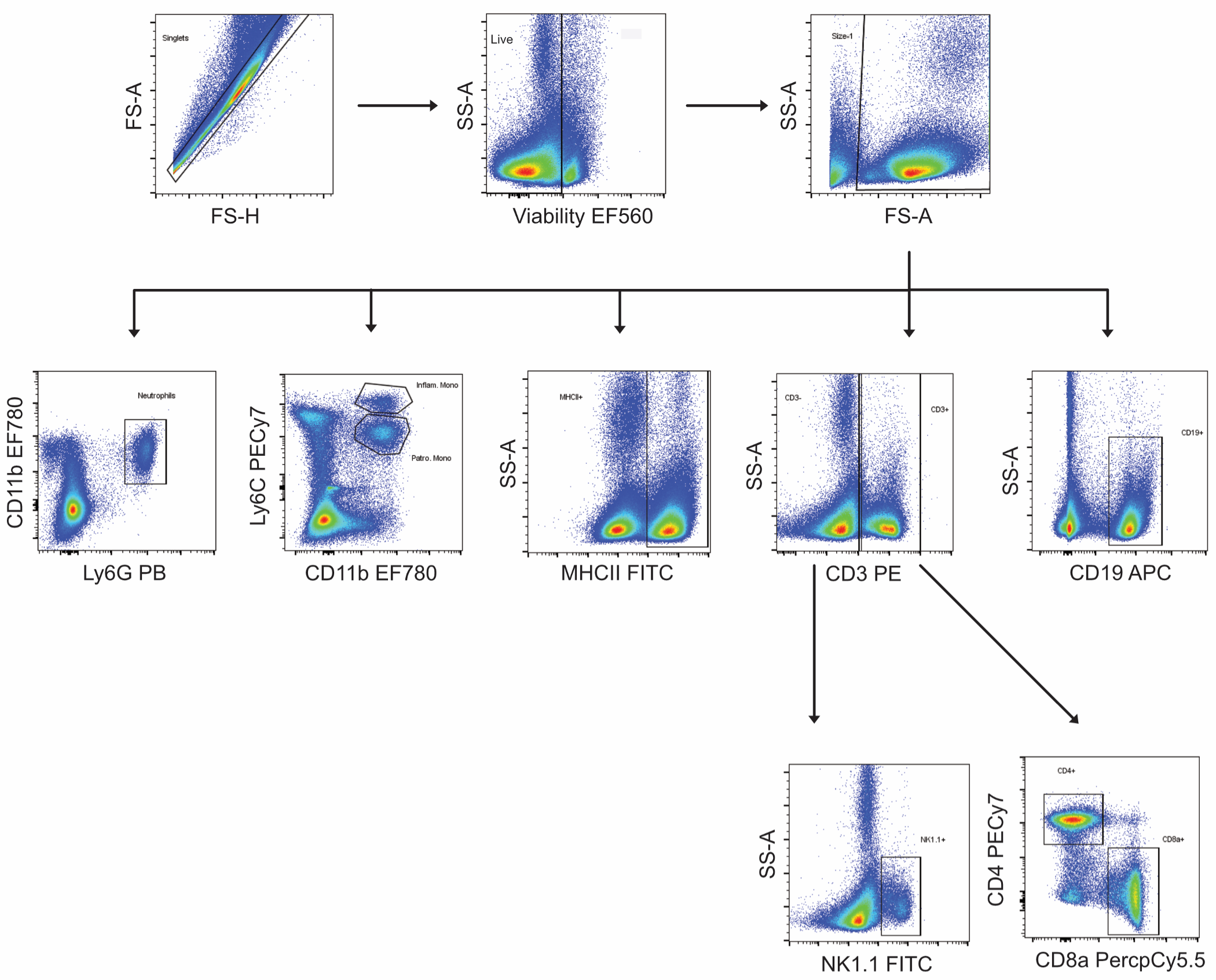

Supplement: Supplementary file 1 — Additional file 1: Fig S1: Gating strategy employed in peripheral immune cell population profiling. C57BL6/J mice were fed a chow diet for 7 days containing CSF1R inhibitor PLX3397 (660 mg/kg). Samples were labeled with mixtures of specific antibodies against immune cell populations and subjected to flow cytometric analysis. Singlets were gated FS-A/FS-H, live mononuclear cells were gated based on SS-A/fixable Viability Dye (eFlour 506), then size gated based on SS-A/FS-A to exclude red blood cells. As indicated in the illustration above, a specific population of choice based on the antibody was identified from the size gate. [file 12974_2023_2924_MOESM1_ESM.tiff]

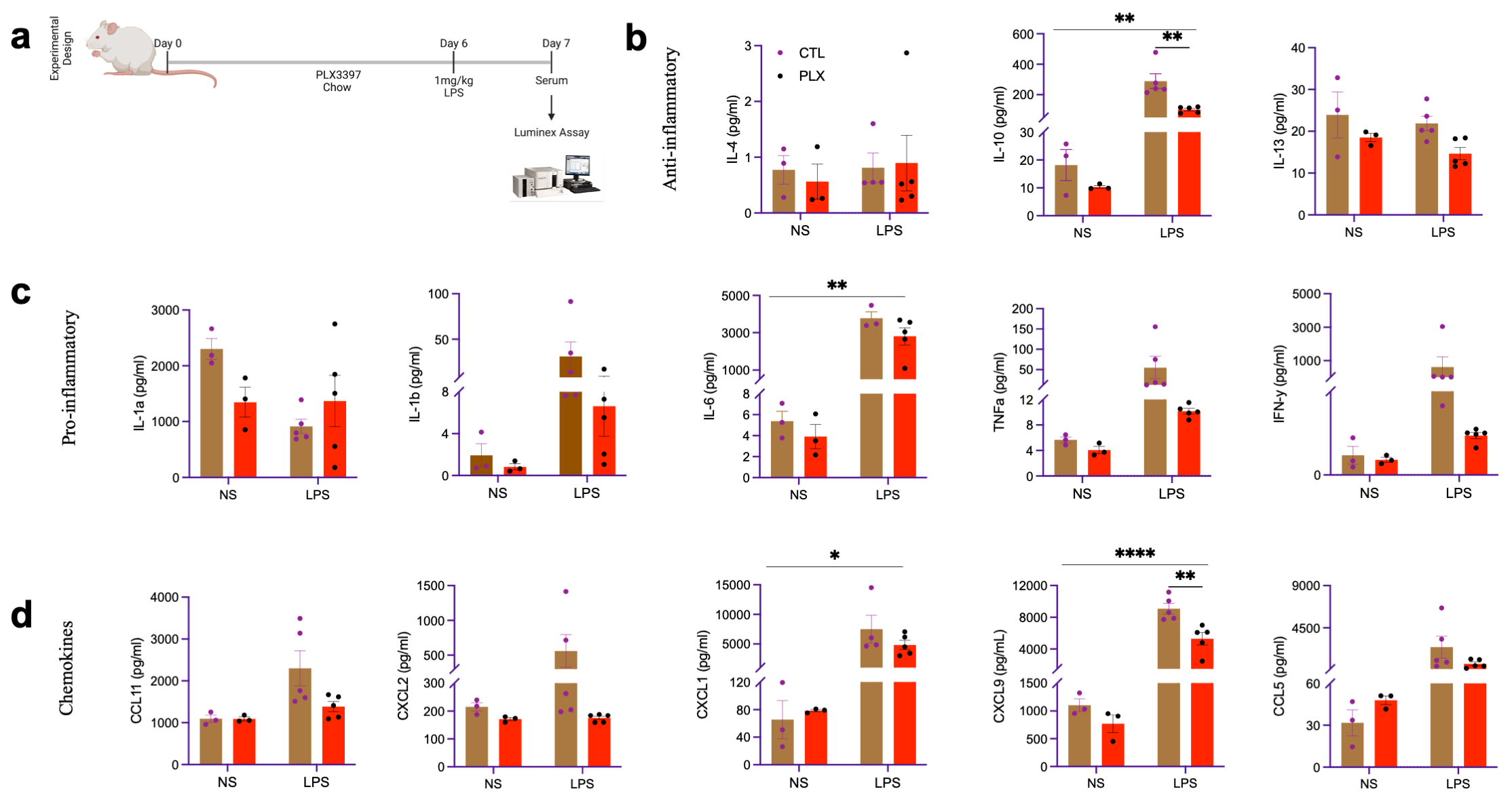

Supplement: Supplementary file 2 — Additional file 2: Figure S2. Seven days of CSF1R inhibition with PLX3397 does not alter serum cytokine/chemokine profile (except IL-10 and CXCL9) 24 h after LPS stimulation. a. Illustration of experimental design showing mice placed on PLX3397 for seven days followed by serum Luminex assay for cytokine profiling. b. serum cytokine anti-inflammatory profile c. serum cytokine pro-inflammatory profile d. serum chemokine profile. Data were analyzed with unpaired Student’s T-test, n = 3–5, and data represented by mean ± SEM, *p < 0.05, **p < 0.01.***p < 0.001, ****p < 0.0001. [file 12974_2023_2924_MOESM2_ESM.tiff]

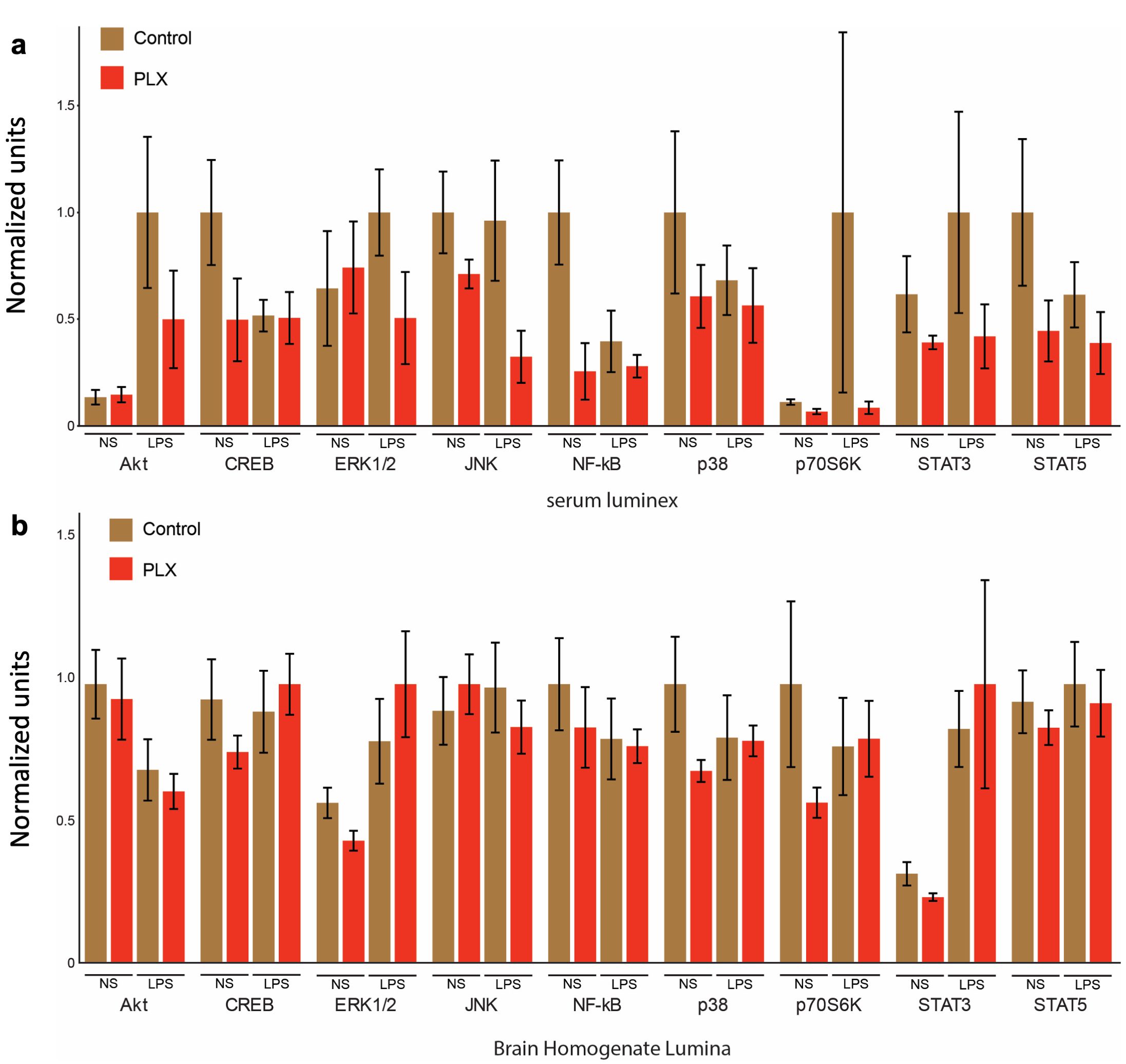

Supplement: Supplementary file 3 — Additional file 3: Figure S3. PLX3397 does not affect cellular kinases in serum and brain homogenate following LPS infection. a. serum cellular kinases pathways b. brain homogenates cellular kinase pathways. Data were analyzed with Student’s unpaired T-tests, n = 5, and data represented by mean ± SEM. [file 12974_2023_2924_MOESM3_ESM.tiff]
